# Evaluation of Acute Flaccid Paralysis in Hamadan, Iran from 2002 to 2009

**DOI:** 10.4178/epih/e2011011

**Published:** 2011-11-16

**Authors:** Jalal Poorolajal, Shadi Ghasemi, Leila Nezamabadi Farahani, Atefeh Sadat Hosseini, Seyyed Jalal Bathaei, Ali Zahiri

**Affiliations:** 1 Research Center for Health Sciences, Department of Epidemiology & Biostatistics, School of Public Health, Hamadan University of Medical Sciences, Hamadan, Iran.; 2 Department of Epidemiology & Biostatistics, School of Public Health, Hamadan University of Medical Sciences, Hamadan, Iran.; 3 Center for Disease Control & Prevention, Deputy of Health Services, Hamadan University of Medical Sciences, Hamadan, Iran.

**Keywords:** Paralysis, Poliomyelitis, Population surveillance, Guillain-Barré syndrome, Iran

## Abstract

**OBJECTIVES:**

To achieve a polio-free certification in Iran, a nationwide active surveillance program for acute flaccid paralysis (AFP) was set up following World Health Organization guidelines. This article describes the results of an eight-year surveillance of AFP in Hamadan, in the west of Iran.

**METHODS:**

A standard set of minimum core variables were collected. All cases of non-polio AFP in children aged <15 years old were reported. Two stool specimens were collected within 14 days of the onset of paralysis.

**RESULTS:**

During the eight-year survey, 88 AFP cases aged <15 years old were reported. About 40% (35/88) of cases were aged ≤5 years, 56% (49/88) were boys, 19 (21.6%) had fever at the onset of paralysis, 74 (84.0%) had complete paralysis within four days of onset, and 22 (24.7%) had asymmetric paralysis. More than one AFP case was detected per 100,000 children aged <15 years old in all years. The risk of AFP in patients aged <5 years old was almost double that of older patients. Guillain-Barré Syndrome was the major leading cause of AFP (66/88). Adequate stool specimens were collected from 85% of AFP patients. All stool specimens were tested virologically, but no wild polioviruses were detected.

**CONCLUSION:**

The active surveillance of non-polio AFP was efficient over the last eight years and exceeded 1.0 case per 100,000 children aged <15 years old. Nonetheless, there was a decreasing trend in the detection of AFP cases during the last two years and should be the focus of the policymakers' special attention, although AFP cases were still above the target level.

## INTRODUCTION

Acute flaccid paralysis (AFP) is often used to describe a sudden onset of flaccid paralysis in one or more limbs in a child aged <15 years old, as might be found with poliomyelitis or other neurologic disorders. Poliomyelitis is an acute viral infection, which can spread directly or indirectly from person to person. The characteristics of this infectious disease range in severity from a non-specific illness to severe flaccid paralysis with permanent disability [1].

In 1998, the World Health Assembly (WHA), which is the directing council of the World Health Organization (WHO), established the target of global polio eradication by the end of 2000 [2]. Four principal strategies were developed for polio eradication, which were proven effective in many countries including (a) high immunization coverage with at least three doses of oral polio vaccine (OPV) in infants aged <1 year; (b) supplemental doses of OPV during national immunization days; (c) mopping-up vaccination in areas or among populations at high risk of poliovirus transmission; and (d) sensitive surveillance and investigation of AFP [3]. Since 1988, the WHA has declared that the "number of countries where polio was endemic decreased from approximately 125 to 6 by the end of 2003" [4].

AFP cases with fever at the onset of paralysis, age <5 years, and asymmetrical paralysis who are unvaccinated are suspected poliomyelitis cases and should be prioritized for investigation [5]. Two key performance indicators are monitored for AFP surveillance: 1) detection of all cases of AFP in children aged <15 years old (target: ≥1 case of non-polio AFP detected annually per 100,000 population aged <15 years old); 2) adequate collection of two stool specimens within 14 days of the onset of paralysis (target: >80% of AFP cases have adequate stool specimens) [6]. AFP surveillance is a 100% sensitive system with poor specificity [7].

The last case of poliomyelitis occurred in Iran due to indigenous transmission of wild poliovirus in 2000. To achieve a polio-free certification in Iran, a nationwide active surveillance program for AFP was set up in the population aged <15 years old following WHO guidelines [8]. The surveillance of AFP began in 1998 under the supervision of the Ministry of Health and Medical Education (MOHME) and continued towards the WHO targets for AFP detection and adequate stool sample collection. This article describes the results of an eight-year surveillance of AFP in Hamadan, in the west of Iran, during the post-polio-free phase and explains the progress made toward polio eradication from 2002-2009.

## MATERIALS AND METHODS

**Figure 1:**
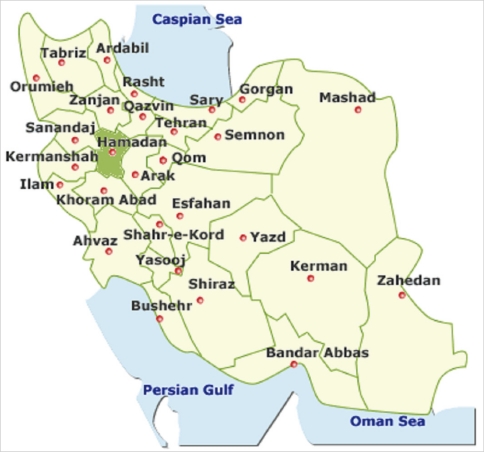
Geographical location of Hamadan province in the Islamic Republic of Iran.

This study reports the results of a surveillance system for non-polio AFP that was conducted according to the WHO recommendations for global eradication of poliomyelitis. In 1998, an active surveillance program for non-polio AFP was set up by the Iranian MOHME according to WHO guidelines using the same strategy throughout the country. All medical universities affiliated the MOHME, including Hamadan University of Medical Sciences, participated in this national survey. Hamadan province is located in the north-west of Iran (Figure 1). According to the survey in 2006, the population of this province was 1,703,267 (2.4% of the total population of Iran) [9].

Based on WHO guidelines, a standard set of minimum core variables that are essential to track the circulation of wild poliovirus, manage national programs and monitor AFP surveillance performance were collected. Three pieces of clinical information were collected from each AFP case and recorded: (a) fever at the onset of paralysis (*Yes/No*); (b) complete paralysis within four days of onset (*Yes/No*); and (c) asymmetric paralysis (*Yes/No*).

All patients with AFP aged <15 years old were detected and reported by phone as soon as possible. All AFP cases considered suspected poliomyelitis and were followed for further evaluation. Two stool specimens or two rectal swab specimens were obtained 24 hours apart from patients during the first 14 days after the onset of paralytic disease. All stool specimens from AFP cases were processed in a WHO-accredited laboratory located in the School of Public Health, Tehran University of Medical Sciences. Virus isolation and typing were performed according to WHO recommended standard methods [10]. AFP cases with inadequate stool specimens were followed up for 60 days. A national expert committee reviewed AFP cases which were clinically compatible with polio with residual paralysis at 60 days and from whom no stool specimens were available or from whom there were negative or inadequate specimens.

The detected cases were divided into three different age groups including <5, 5 to 9, and 10 to 14 years old. Then, in order to estimate the mean annual incidence rate of non-polio AFP per 100,000 population aged <15 years old, the cases of each age group were considered as the nominator and the relevant population as the denominator using the Poisson regression model. Poisson regression analysis was performed at a 95% significant level using the statistical software Stata version 10 (StataCorp, College Station, TX, USA).

## RESULTS

During the eight-year survey, from 2002 to 2009, 88 AFP cases were reported. The patients were aged <15 years with a mean age of 6.8 years. About 40% (35/88) of AFP cases aged ≤5 years, 56% (49/88) were boys, and 24% (21/88) were reported in 2007 (Table 1).

Of the 88 AFP cases, 19 (21.6%) had fever at the onset of paralysis, 74 (84.0%) had complete paralysis within four days of onset, and 22 (24.7%) had asymmetric paralysis.

According to the Poisson regression analysis, the risk of AFP in patients aged <5 years old was almost double that of patients aged 5-9 and 10-14 years old (p=0.030 and p=0.012, respectively). However, there was no statistically significant differences between the risk of AFP among girls and boys (p=0.414) (Table 2).

**Figure 2:**
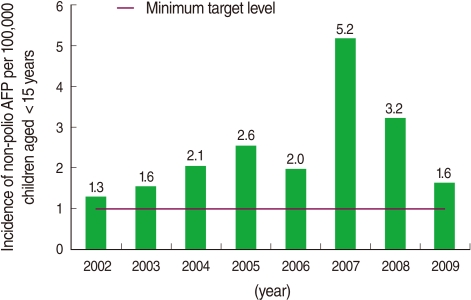
Annual incidence rate of non-polio AFP per 100,000 population aged <15 years old in Hamadan, Iran (2002 to 2009). AFP, acute flaccid paralysis.

The results of a complete virologic investigation of AFP cases from 2002 to 2009 are presented in Figure 2. According to these findings, the surveillance system could achieve and even exceed the expected minimum level defined by WHO as the target of detecting more than one non-polio AFP case per 100,000 population aged <15 years old in all years.

**Figure 3:**
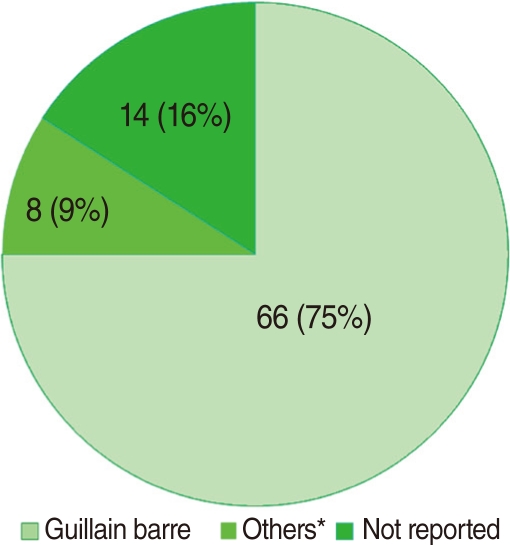
Differential diagnosis of non-polio acute flaccid paralysis among the population aged <15 years old in Hamadan, Iran (2002 to 2009). ^*^others includes transverse myelitis, multiple sclerosis, nerve lesions, epilepsy, acute viral infection, and cerebrospinal tumor.

Data on the differential diagnosis of AFP are presented in Figure 3. Guillain-Barré syndrome was among the major leading cause of AFP (66 out of 88 cases). Eight (9%) AFP cases were categorized as "others" including transverse myelitis, multiple sclerosis, nerve lesions, epilepsy, acute viral infection, and cerebrospinal tumors. The final clinical diagnosis was not reported for 14 (16%) cases, although laboratory findings refuted the probability of poliomyelitis.

**Figure 4:**
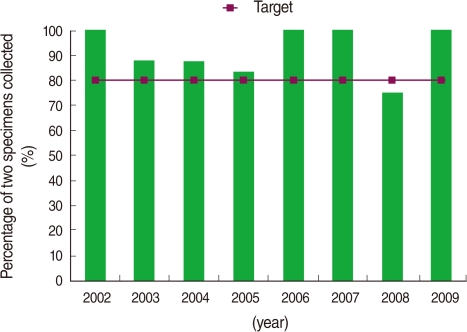
Percent of non-polio acute flaccid paralysis cases among the population aged <15 years old whose stool specimens were collected during the first or second week after the onset of paralytic disease in Hamadan, Iran (2002 to 2009).

The information on the percentage of AFP cases with two adequate stool specimens is presented in Figure 4. Accordingly, at least two stool specimens or rectal swab specimens were collected from all patients. The two stool specimens were obtained 1-5 days apart (1.2 days on average). The specimens of almost 85% (75) of the cases were collected during the first or second week after the onset of paralytic disease and the others were collected during the third week. All stool specimens were tested virologically, but no wild polioviruses were detected.

## DISCUSSION

The present study represents a comprehensive province-based survey of non-polio AFP in Iran, which reports the results of an eight-year AFP surveillance program in Hamadan province, in the west of Iran, from 2002 to 2009. There is a nationwide health network in Iran, which covers all parts of the county, even small, remote villages. It is obligatory for all health workers throughout the country to report and follow up any cases of AFP. With such a widespread and consolidated health system, it is rare that a case of AFP is missed.

Detecting and investigating all cases of non-polio AFP in the population <15 years old are among the most criteria for poliomyelitis-free certification. In polio-free regions, the incidence of AFP is expected to be at least one or more per 100,000 children aged <15 years old [6]. The results of this study indicated that active surveillance of AFP was efficient over the past eight consecutive years and even exceeded the WHO-established minimum non-polio AFP rate. In addition, the second criteria of AFP surveillance, adequate collection of two stool specimens within 14 days of the onset of paralysis in at least 80% of detected AFP cases [6], was achieved efficiently as well.

National surveys in Pakistan and Afghanistan have reported relatively higher AFP rates compared to the results of present study, but a lower percentage of adequate stool specimens. Accordingly, non-polio AFP rate for Pakistan in 2003 was 3.0 per 100,000 children aged <15 years old. The percentage of AFP cases with adequate stool specimens was 89% and 90% in 2003 and 2004, respectively. Non-polio AFP rates in Afghanistan were 4.0 per 100,000 in children aged <15 years old in 2003 and 4.2 in 2004. The percentage of patients with adequate stool specimens was 88% in 2003 and 93% in 2004 [11].

Detection and investigation of AFP cases increased over time from 2002 to 2007, but decreased thereafter. This may be a serious alarm for the AFP surveillance system, particularly if this decreasing trend continues in subsequent years. Although AFP cases were above the target level, this concern should be the focus of policymakers' special attention.

The frequency of AFP was higher among boys than among girls (49 versus 39, respectively); nonetheless, the mean annual incidence rate of AFP per 100,000 boys and girls was not statistically significant (p=0.414). D'Errico et al. conducted a survey in Italy and reported 15 AFP cases among boys compared to 12 AFP cases among girls [10]. Oostvogel et al. [12] in the Netherlands reported 34 AFP cases in boys in comparison with 18 AFP cases in girls.

Guillain-Barré syndrome is known as the major cause of non-polio AFP, particularly after the global eradication of poliomyelitis [13]. This syndrome was the most common cause of non-polio AFP cases and comprised 75% of all causes of AFP cases in the present study. A study conducted by Davarpanah et al. in 2008 [14] in Shiraz province, in central Iran, reported a similar result. They indicated that Guillain-Barré Syndrome was the main leading cause of AFP in 66% of the patients.

As mentioned in the introduction section, to achieve polio-free certification in Iran, a nationwide active surveillance program for AFP was set up simultaneously with same methods for the target population in all provinces of Iran following WHO guidelines. The surveillance of AFP began in 1998 under the supervision of the Ministry of Health and Medical Education and continued towards the WHO targets for AFP detection and adequate stool sample collection. This paper is part of the results of this nationwide surveillance of non-polio AFP. In addition, Hamadan is not a border province and hence cross-border population movements are rare if they occur at all. Accordingly, it is expected that this survey may represent the profile of the national survey in Iran over the last eight years and its results may be generalized to the vast majority or at least most parts of the country, although diversity between different provinces is possible.

This study had a number of limitations. First, the data on AFP cases reported from 2002 to 2006 were disorganized, incomplete, and illegible and hence had to be compared with the data recorded based on telephone reports. Second, the final clinical diagnosis of 14 AFP cases was not recorded. Furthermore, the results of the decision made by the national expert committee who reviewed AFP cases which were clinically compatible with polio with residual paralysis at 60 days were not recorded for a number of patients.

Despite its limitations, the current study may have a number of implications on health care policy. First, the performance of the non-polio AFP surveillance program was evaluated in the target population for eight consecutive years. Second, the association between male gender and AFP was clearly indicated. Furthermore, Guillain-Barré Syndrome was found to be the main cause of AFP among children aged <15 years old.

The results of this survey indicate that the active surveillance of non-polio AFP was efficient over the past eight years and exceeded 1.0 case per 100,000 children aged <15 years old, the WHO-established minimum non-polio AFP rate. Furthermore, the percentage of patients with AFP from whom adequate stool specimens were collected was above the minimum target level of 80%. Nonetheless, there was decreasing trend in the detection of AFP cases over the last two years, although AFP case numbers were still above the target level. However, this trend may continue in subsequent years and should be the focus of policymakers' special attention. We also found Guillain-Barré Syndrome was the most common cause of non-polio AFP cases, particularly in boys.

## Figures and Tables

**Table 1:**
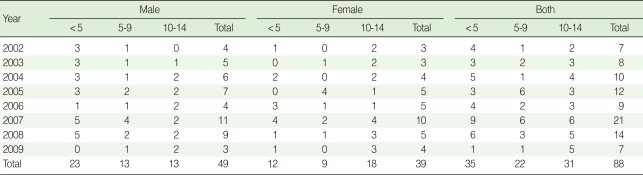
Distribution of non-polio acute flaccid paralysis by year, sex and age group

**Table 2:**
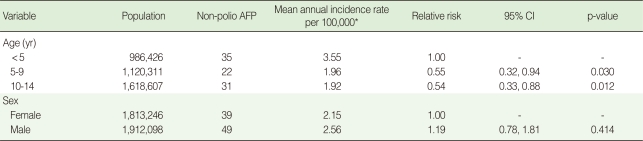
Mean annual incidence rate of non-polio acute flaccid paralysis (AFP) per 100,000 under 15 years old children by age and sex using Poisson regression analysis

CI, confidence interval. ^*^In order to estimate the mean annual incidence rate of non-polio AFP per 100,000 population aged <15 years old, the cases of each age group were considered as the nominator and the relevant population as the denominator.
